# Imaging-based techniques for ablation zone definition and volumetry after laser interstitial thermal therapy (LITT) for intracranial lesions: a systematic review

**DOI:** 10.1007/s00701-025-06666-6

**Published:** 2025-10-08

**Authors:** Céline L. G. Neutel, Thomas M. Putinela, Maroeska M. Rovers, Pierre A. Robe, Mark ter Laan, Christiaan G. Overduin

**Affiliations:** 1 Department of Neurosurgery, Radboud University Medical Center, Nijmegen, The Netherlands; 2 Department of Medical Imaging, Radboud University Medical Center, Nijmegen, The Netherlands; 3 Department of Neurosurgery, University Medical Center Utrecht, Utrecht, The Netherlands

**Keywords:** LITT, Laser ablation, Laser interstitial thermal therapy, Imaging, Extent of ablation, Ablation volume

## Abstract

**Purpose:**

MRI-guided laser interstitial thermal therapy (LITT) is a minimally invasive technique for treating intracranial pathologies. Although the extent of ablation appears prognostically relevant, standardized imaging methods for post-LITT ablation zone measurements are lacking. This systematic review evaluates imaging-based approaches used to measure the ablation zone in patients undergoing LITT. As effect assessment is an integral part of the technique, this study aims to support the development of standardized imaging-based outcome metrics.

**Methods:**

A systematic literature search was conducted in PubMed and Embase (March 15, 2024; updated April 2, 2025). Studies were included if they reported imaging-based methods for determining ablation extent or volume after LITT; studies without methodological detail, non-original research, or non-human studies were excluded. Study selection, data extraction, and risk of bias assessment (Newcastle–Ottawa Scale) were conducted independently by multiple reviewers.

**Results:**

A total of 77 studies (2,312 patients) were included. Most studies (82%) were retrospective case series, with 74 (96%) categorized as having moderate risk of bias. All studies utilized MRI to assess post-LITT ablation volume. Conventional MRI sequences were used in 65 studies (84%), among which 54 (83%) used contrast-enhanced imaging. Forty-six studies (60%) reported a single time-point volume assessment. Of the 60 studies using contrast-enhanced imaging, 50% specified inclusion or exclusion of the enhancing rim.

**Conclusion:**

Our results show considerable variation and underreporting regarding rim inclusion, measurement timing, and volume definitions. Standardized imaging protocols, covering timing, modalities, and rim handling, are essential to improve LITT research and outcomes. We propose four recommendations to guide future reporting of imaging methods.

**Clinical trial number:**

Not applicable.

**Supplementary Information:**

The online version contains supplementary material available at 10.1007/s00701-025-06666-6.

## Introduction

Laser interstitial thermotherapy (LITT) is a minimally invasive therapeutic modality used for treating various intracranial pathologies, including high-grade gliomas, epilepsy, and radiation necrosis [68, 71]. LITT involves the precise navigation and positioning of a laser probe within the lesion, followed by the ablation of the targeted tissue under MRI guidance.

Owing to its minimally invasive nature, LITT is particularly interesting for patients suffering from deep-seated lesions or lesions located in or near eloquent areas of the brain, such as (high-grade) tumors in the basal ganglia or hypothalamic hamartomas in epilepsy patients [15, 68, 75]. The ability to reach and treat lesions in challenging locations represents a significant advancement in neurosurgical interventions, offering the possibility of cytoreduction where it was previously unattainable [68]. By using real-time MR-thermometry, the ablation volume can be tailored where maximally safe ablation of the target lesion is targeted while minimizing damage to the surrounding healthy brain tissue [15].

Similar to the extent of resection in brain tumor surgery, the extent of ablation (EOA), defined as the percentage of the ablated region overlaying the target region, has been associated with improved prognosis in LITT in some retrospective studies [40, 79]. The post-LITT ablation zone is typically visualized on immediate contrast-enhanced T1-weighted MRI as a hypo- or isointense central necrotic core, surrounded by a hyperintense, contrast-enhancing peripheral rim [31]. Nevertheless, particularly in high-grade glioma cases, the accurate delineation of the ablation volume may be challenging as the typical rim enhancement in the periphery of the ablation zone following LITT may be difficult to distinguish from the rim-enhancing appearance of pre-existing vital tumor. Alternatively, thermometry data and ‘surgeon estimate’ are used to derive ablation zone volumes or estimate the EOA [19, 40, 77]. However, a standardized or well-validated method for ablation zone definition or volumetry after LITT, and consequently deriving the EOA, is currently lacking.

Given that the assessment of the effect is an integral part of the technique, this systematic review aims to evaluate the imaging-based methods used to define and measure the ablation zone in patients undergoing LITT for intracranial lesions. Specifically, the types of modalities used, timing of post-procedural imaging, and volumetric assessment techniques were examined. By synthesizing and analyzing current practices, the review aims to inform the development of standardized imaging-based outcome measures following LITT.

## Materials and methods

This systematic review was reported according to the PRISMA guidelines [66]. The PRISMA checklist can be found in the supplementary materials (Supplementary Item 1). The protocol for this systematic review has been registered in PROSPERO (CRD42024515809).

### Literature search

We performed a systematic literature search in PubMed and Embase for all studies reporting on imaging-based assessment of ablated volumes or percentages of ablation after LITT in intracranial lesions on March 15, 2024. Search terms included combinations of *“laser therapy”* or related terms such as *“Laser interstitial thermal therapy”, “MRgLITT”,* and *“SLA”,* in conjunction with terms related to intracranial lesions, such as *“brain tumor”, “glioblastoma”,* and *“epilepsy”.* The search phrase was kept broad in order to find all potentially relevant articles. The full search strategy is available in the supplementary materials (Supplementary Item 2). The search was repeated on April 2, 2025, resulting in the inclusion of two additional studies.

Duplicate articles were eliminated using Endnote X9 bibliographic database (Clarivate Analytics, Boston, MA, USA). After duplicate removal the articles were uploaded in Rayyan [65]. Selection of relevant articles was first done based on title and abstract by two reviewers (CN and TP). Discrepancy in the selection of articles was resolved by a third reviewer (CO). After title and abstract screening, the full texts of the remaining articles were sought and screened. Again, discrepancy in the selection of articles between the two reviewers was resolved by the third reviewer. To ensure the completeness of our search strategy, we conducted citation tracking in addition to systematic database searches. Using Citationchaser [28] we screened the reference lists of the 77 included studies (backward citation tracking) as well as studies that cited the included articles (forward citation tracking) to identify any potentially relevant additional studies, but no additional studies met the inclusion criteria.

Data extraction was done by one author (CN) and was checked on a sample basis by a second reviewer (TP). Quality assessment was done independently by two authors (CN and TP). The risk of bias was evaluated using the validated Newcastle–Ottawa Quality Assessment Scale as advised by the Cochrane Collaboration [30, 88]. Any discrepancies were resolved through consensus and if consensus could not be reached, a third reviewer (MR) resolved any discrepancy.

### Criteria for selecting studies

We included all studies that reported imaging-based assessment of percentage of ablation, extent of ablation or volume of the ablation zone following LITT in intracranial lesions in human patients and the method(s) for defining these outcomes. Studies that reported only the percentage of ablation/EOA or the volume of the ablation zone, without detailing the methods used to determine these outcomes, were excluded from this systematic review. Although this was not initially specified as an exclusion criterion in the protocol registered with PROSPERO, the decision to introduce this criterion was made to better align with the primary aim of our review, which was to examine the methods used for volume determination. While it is important to acknowledge that some studies report postoperative volumes without providing methodological details, such studies did not align with the objective of this review. Therefore, to ensure clarity and focus in the results, this exclusion criterion was added.

Furthermore, articles were excluded if they did not report on either percentage of ablation/EOA or volume of the ablation zone, but instead focused solely on other outcomes such as survival, complications, or progression-free survival. Additional exclusion criteria included: (1) systematic reviews, (2) conference abstracts, (3) commentaries, (4) letters to the editor, (5) in-vitro and animal studies, (6) case reports with fewer than 3 patients, and (7) studies based on the same dataset. No studies were excluded based on this final exclusion criterion. Studies investigating the same patient population but describing different methods for ablation volume determination were included.

### Data extraction

Data was extracted from text, figures and tables.

The following data was extracted if mentioned in the article:

- Study characteristics: title, authors, country, year of publication, study design, disease(s) studied, population studied
- Lesion characteristics oncology: lesion type(s)/treatment etiology, volume before treatment
- Ablation characteristics: LITT device used, number of trajectories, number of ablations, definition of ablated volume, ablation percentage/EOA and/or ablated volume
- Measurement of ablation zone: modality, software used for image processing, timing of measurement, method of segmentation, definition of ablation borders, method of calculation of ablation percentage or ablation volume

### Data analyses

The data are presented descriptively and summarized in tables to provide a comprehensive overview. All outcomes were extracted from all studies. If an outcome was not reported in a study, it was recorded as “not reported (N.R)”. If the original paper specified whether the reported value was a median or mean and explicitly stated the measure of dispersion, this information was retained in the table of this systematic review. If either the central tendency or the measure of dispersion was not specified, this was left open in the table. Studies without a control group were classified as case-series, consistent with the categorization by the Cochrane Collaboration [2]. Studies were classified as retrospective when the research question was formulated after data collection. A study was classified as prospective when clear inclusion and exclusion criteria were established prior to data collection to address the specific research question.

Data are presented in tables categorized by LITT indication (oncology, epilepsy, or both), with each study listed separately. Post-LITT measurements are displayed in the tables, as these served as the primary criterion for selecting articles and for presenting the methods and terminology used to define post-LITT outcomes. In cases where pre- and/or post-LITT measurements were reported both per patient and as a median or mean, only the median or mean values were included in this review. Papers that provided the median or mean alongside individual patient measurements are marked with an asterisk. For papers that reported only individual patient measurements, these measurements were included in this review.

The imaging modality used for the calculation of the ablation zone was recorded. In cases where MRI imaging was employed, it was categorized as either conventional MRI sequences or thermal MRI sequences. Additionally, for studies utilizing contrast-enhanced MRI, we recorded whether the contrast-enhancing rim, typically seen as a hyperintense border surrounding the ablation zone on post-contrast MRI, was included in the volume calculation.

Due to the heterogeneity of the studies, further data synthesis could not be performed.

### Risk of bias assessment

The Newcastle–Ottawa Scale (NOS) was used to assess the risk of bias assessment, as it was the most suitable tool for evaluating the included studies, despite the majority being retrospective case-series. For our systematic review, we concentrated on the methods used to determine the ablation zone, which is often not the primary outcome of the included studies. In the risk of bias assessment, we evaluated the overall quality of the study, rather than focusing solely on how well the study was designed to measure our specific outcome of interest. This approach is consistent with standard practice for conducting a risk of bias assessment using the NOS. For studies without a control group but where statistical analyses appropriately accounted for relevant confounders, one point was awarded for "Comparability." In the “Outcome” section, no points were assigned if the length of follow-up or the completeness of follow-up/loss to follow-up was unclear or inadequately reported.

## Results

### Search

**Fig. 1:**
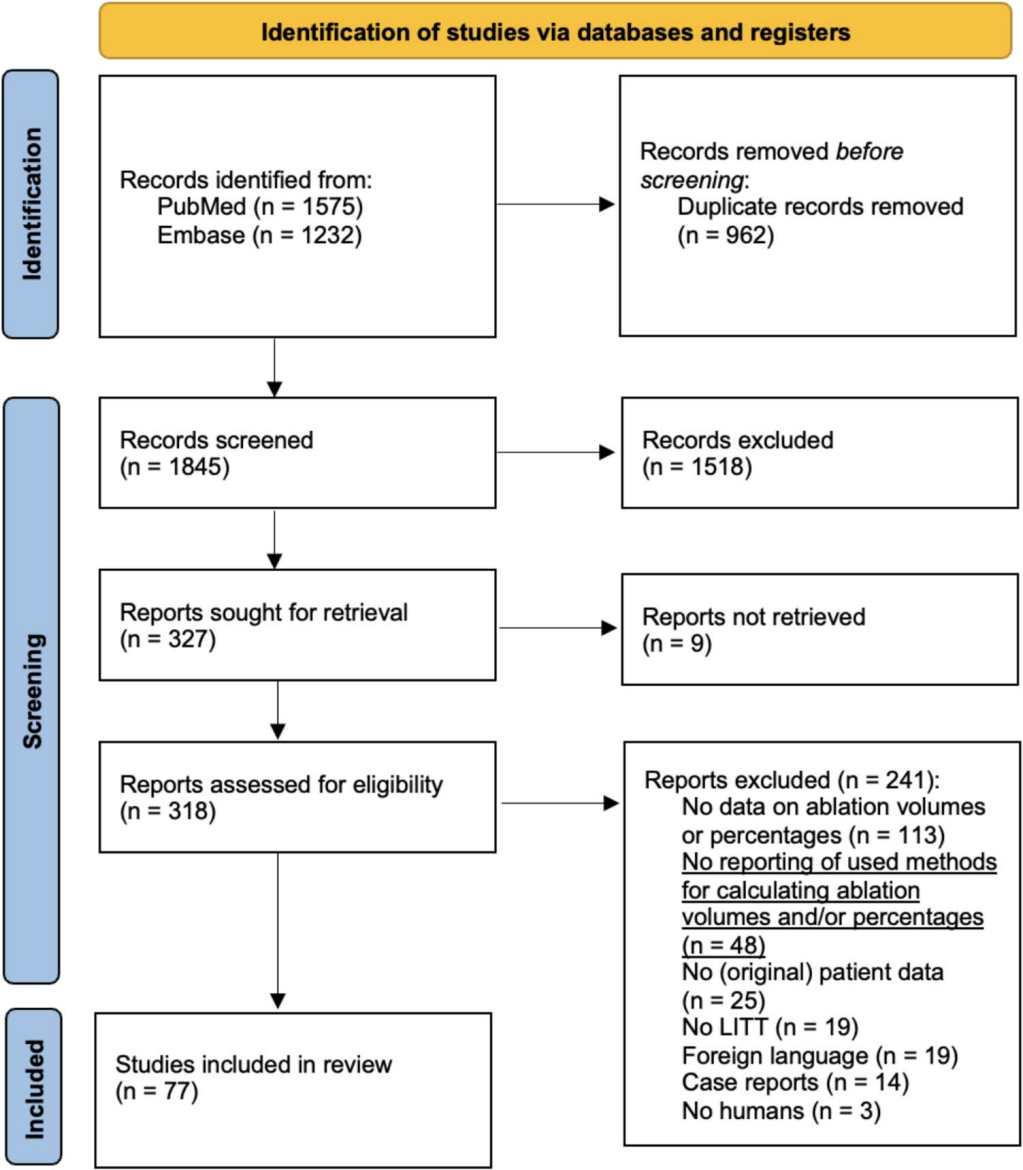
PRISMA flowchart

From the initial search, 2,807 articles were identified. After removing duplicates, 1,845 articles remained and were screened. Title and abstract screening resulted in 327 articles. Of these, 318 full texts were retrieved and screened. The full texts of 9 articles could not be retrieved because the full texts could not be found online. Ultimately, 77 articles were included in this systematic review [1, 4–14, 16–18, 21–27, 29, 32–64, 69, 70, 72, 74–78, 80–87, 89–93] (Fig. 1). Notably, during the screening process for this review, it was found that 48 papers provided outcomes for ablation volume or ablation percentage but did not explain any aspect of the methods used to obtain these volumes/percentages in the paper (see the underlined section in the PRISMA flowchart, Fig. 1).

### Study characteristics and risk of bias

The 77 included studies were published between 1994 and 2024. The majority of studies (*n* = 63) were retrospective case-series. The remaining studies comprised 9 prospective case series and 5 retrospective cohort studies. The sample size per study varied from 4 to 313 patients, with a median of 20 patients. A total of 39 studies investigated oncological indications for LITT, 33 focused on epilepsy, 4 reported both oncological and epilepsy indications, and 1 study examined LITT for oncological, epilepsy, and pain indications. The study characteristics and results are presented in separate tables for each treatment indication. The results for studies focusing on oncology, including radiation necrosis, are shown in Table 1, those for studies focused on epilepsy in Table 2, and studies addressing multiple or other indications are presented in Table 3. The full data extraction table is available from the corresponding author upon request.

**Table 1:** Study characteristics and outcomes for studies focusing on oncology indications

Author	Study design	Sample size	Pre-operative/target volume or area	Definition of ablated volume	Post-LITT measurement of ablated volume
Ashraf (2020)	Retrospective case series	58	2.24 ± 0.21 cm^3^ (mean, SE)	24-h post ablation volume	3.92 ± 0.28 cm^3^ (mean, SE)
Bartlett (2023)	Prospective case series	17	N.R	Percentage of ablation	95.9 ± 7.3% (median)*
Bastos (2020)	Retrospective case series	61	4.02 cm^3^ (0.2—26.3 cm^3^) (median, range)	Ablation volume	7.72 cm^3^ (1.52—26.72 cm^3^) (median, range)
Beaumont (2018)	Retrospective case series	15	18.7 ± 16.9 cm^3^ (mean)*	Percentage coverage yellow and blue isodose	Yellow: 95.4 ± 7.7% (yellow), blue: 91.2 ± 11.5% (means and SDs)*
Beechar (2018)	Retrospective case series	36	ceT1: 5.05 cm^3^ (0.54—23.31 cm^3^)//FLAIR: 43.36 cm^3^ (3.09—233.01 cm^3^) (medians, ranges)*	Post-LITT volume	Post-LITT: ceT1: 7.23 cm^3^ (0—42.5 cm^3^), FLAIR: 43.51 cm^3^ (8.88—218.5 cm^3^)//0–90 days: ceT1: 7.7 cm^3^ (1.72—38.76 cm^3^), FLAIR: 37.13 cm^3^ (3.48—244. 23 cm^3^)//90–180 days: ceT1: 5.15 cm^3^ (1.53—39.93 cm^3^), FLAIR: 26.06 cm^3^ (0.50—311 cm^3^)//180–270 days: ceT1: 3.68 cm^3^ (1.28—48.31 cm^3^), FLAIR: 31.68 cm^3^ (1.60—348.75 cm^3^)//270–356 days: ceT1: 2.69 cm^3^ (0.38—14.57 cm^3^), FLAIR: 4.68 cm^3^ (1.36—33.98 cm^3^) (medians, ranges)*
Borghei-Razavi (2018)	Retrospective case series	8	4.35 cm^3^ (mean)*	Percent tumor coverage and 1-day postoperative ablation volume	98% (90–100%) (mean, range) Residual: 9.64 cm^3^ (mean)*
Carpentier (2012)	Prospective case series	4	5542 mm^3^, 2145mm^3^, 1224 mm^3^, 1020 mm^3^	Ablated volume	8864 mm^3^, 3591 mm^3^, 1767 mm^3^, 2535 mm^3^, 1978 mm^3^
Carpentier (2011)	Prospective case series	7	N.R	Relative lesion size	26% (0—124%) (mean, range)*
Chaunzwa (2017)	Retrospective case series	30	7.6 cm^3^ (0.6—38.9 cm^3^) (median, range)	Percentage cell death and percentage protein denaturation	Cell death: 96% (41—100%) // Protein denaturation: 98% (80—100%) (medians, ranges)
Dadario (2022)	Retrospective case series	35	3.80 ± 3.13 cm^3^ (mean, SD)	Percentage volume ablation and residual tumor volume	91 ± 13% (mean, SD) Residual: 0.45 cm^3^ (0.77 cm^3^) (mean, SD)
Daggubati (2023)	Retrospective cohort	25 (of which 11 LITT)	29.96 cm^3^ (mean, total group), 27.15 cm^3^ (mean, LITT group)	Postoperative volume, extent of treatment and postoperative residual volume	32.28 cm^3^, 73.84% (means) Residual: 8.84 cm^3^ (mean)
Eichberg (2018)	Retrospective case series	4	3.35 cm^3^ (1.1—7.2 cm^3^) (average, range)*	Percentage of lesion ablated	97.1% (88.2%- 100%) (average, range)*
Fadel (2022)	Retrospective cohort	40 (of which 17 LITT)	4.7 ± 3.4 cm^3^ (mean, SD), 4.37 cm^3^ (1.77—7.02 cm^3^) (median, IQR)	Extent of ablation (EOA)	90.7 ± 16.1% (mean, SD), 100% (89%, 100%) (median, IQR)
Gurses (2024)	Retrospective case series	313	11.32 ± 19.9 cm^3^ (mean, SD)	Extent of ablation (EOA) and postoperative volume	190.6 ± 198.2% (mean, SD)//15.8 ± 45.6 cm^3^ (mean, SD)//7 cm^3^ (3.3—14.6 cm^3^) (median, IQR)
Haskell-Mendoza (2024)	Retrospective case series	56	N.R	Contrast-enhancing Lesion Volume (CeVL)	CeVL increase from baseline: 68.3% (35.1—109.2%) (median, IQR)*
Kahn (1994)	Retrospective case series	8	18 mm, 28 mm, 27 mm, 25 mm, 29 mm, 20 mm, 20 mm, 34 mm (diameter)	Percentage of tumor comprised by laser-induced lesion	101% (64—130%) (mean)*
Kaisman-Elbaz (2023)	Retrospective case series	56	11.7 cm^3^ (2.2—35.3 cm^3^) (median, 95% CI)	TDT-line coverage (yellow and blue) and residual tumor volume	Yellow TDT line: 99.4% (76—100%)//blue TDT line: 96.1% (62—100%) (medians and 95%CIs) Residual outside blue TDT line: 0.3 cm^3^ (0—9 cm^3^) (median, 95%CI)
Luther (2021)	Retrospective case series	17	2.0 ± 1.5 cm^3^ (mean)	Lesion volume	IPA: 4.8 ± 2.2 cm^3^//Last FU: 1.7 ± 0.9 cm^3^ (median or mean)
Luther (2020)	Retrospective case series	20	8.474 cm^3^ (0.885—31.69 cm^3^) (mean, range)	Post-LITT lesion volume for radical volumetric ablation	9.054 cm^3^ (mean). Other categories (subtotal diametric/volumetric ablation and total diametric/volumetric ablation) not reported
Maraka (2018)	Retrospective case series	8	26.54 cm^3^, 64.74 cm^3^, 101.48 cm^3^, 200.52 cm^3^, 36.86 cm^3^, 14.55 cm^3^, 32.12 cm^3^, 11.03 cm^3^	Tumor volume post-LITT	27.48 cm^3^, 93.94 cm^3^, 151.71 cm^3^, 221.05 cm^3^, 87.76 cm^3^, 73.29 cm^3^, 44.82 cm^3^, 82.01 cm^3^
Merenzon (2024)	Retrospective case series	250	Q1: 5.7 cm^3^, Q2: 8.5 cm^3^, Q3: 10.5 cm^3^, 11.9 cm^3^ (means) *(estimates from table!)*	Extent of ablation (EOA) (in figure)	Q1: 225%, Q2: 195%, Q3: 200%, Q4: 160% (means) *(estimates from figure!)*
Merenzon (2023)	Retrospective case series	33	3.6 ± 3 cm^3^ (mean ± SD)	Extent of ablation (EOA)	162 ± 131% (mean, SD)
Merenzon (2022)	Retrospective cohort	22 (of which 7 LITT)	16.99 ± 9.23 cm^3^ (LITT group, average)	Extent of ablation (EOA) (subdivided)	1 × radical (> 200%), 5 × total (100–200%), 1 × subtotal (< 100%)
Missios (2014)	Retrospective case series	11	12.40 cm^3^ (2.2—25.4 cm^3^) (median, range)	TDT-lines coverage and residual tumor volume not covered by TDT-lines (yellow and blue)	Blue TDT-line: 94% (28–100%)//yellow: 99% (34–100%) (means, ranges) Residual by blue TDT-line: 0.86 cm^3^ (0—9.0 cm^3^), by yellow TDT-line: 0.09 cm^3^ (range 0—8.3 cm^3^) (medians, ranges)
Mohammadi (2014)	Retrospective case series	34	10.13 cm^3^ (0.7—49.9 cm^3^) (median, range)	TDT-lines coverage and residual tumor volume not covered by TDT-lines (yellow and blue)	Blue: 91% (28–100%)//yellow: 98% (34–100%) (medians, ranges) Residual by blue TDT-line: 0.66 cm^3^ (0—22.5 cm^3^), by yellow TDT-line: 0.19 cm^3^ (0—15.5 cm^3^) (medians, ranges)
Muir (2022)	Retrospective case series	20	11.34 cm^3^ (median)*	Gross Total Ablation	Gross Total Ablation (GTA, defined by less than 5% of the tumor volume not covered by the ablation radius): 50%, no GTA: 50%
Muir (2022)	Retrospective case series	13	9.44 cm^3^, 13.7 cm^3^, 7.68 cm^3^, 0.74 cm^3^, 21.10 cm^3^, 55.4 cm^3^, 0.04 cm^3^, 22.7 cm^3^, 6.6 cm^3^, 0.38 cm^3^, 2.38 cm^3^, 7.33 cm^3^	Extent of Ablation (EOA)	First LITT: 79%//Second LITT: 70%//Third LITT: 83% (averages)
Murayi (2020)	Retrospective case series	13	12.0 cm^3^ (mean), 13.77 cm^3^ (1.67—30.3 cm^3^) (median, range)*	TDT coverage by yellow and blue line, tumor volume treated and tumor volume shrinkage	Yellow: 98%, blue: 95% (medians)//Tumor volume treated: 12.0 cm^3^ (1.6—30.3 cm^3^) (mean, range)//Percentage shrinkage (2/3 months post-op MRI): N.R., 47.8%, 73.1%, 48.0%, 29.5%, N.A., N.A., 63.7%, N.R., 84.2%, 25.8%, 76.4%, N.A. (early death)
Patel (2013)	Retrospective case series	16	4.12 ± 4.69 cm^3^ (mean)*	Lesion volumes (in figure)	IPA: 7.8 cm^3^//24 h postop: 4.9 cm^3^//first FU: 4.3 cm^3^ (averages) (estimates from figure!)*
Patel (2015)	Retrospective case series	4	8.4 ± 6.3 cm^3^ (average)*	Ablated volume and percentage ablated volume	4.4 ± 2.7 cm^3^//64.4 ± 23.5% (average)*
Rammo (2018)	Prospective case series	10	1.62 ± 0.31 cm^3^ (mean ± SE)	Ablation volumes and percentage ablated	IPA: 3.58, 0.75 cm^3^//1-2w PO: 6.93, 1.64 cm^3^//1-2 m PO: 5.30, 1.34 cm^3^//3-4 m PO: 2.50, 0.96 cm^3^//5-6 m PO: 4.16, 1.06 cm^3^//> 6 m PO: 1.11, 0.76 cm^3^ (means, SE)//86.4% (average)
Rao (2014)	Prospective case series	15	3.66 cm^3^ (0.46—25.45 cm^3^) (mean, range)*	Increase in volumetric size	278% (112—771%) (average, range)*
Reese (2024)	Retrospective case series	61	19.6 cm^3^ (mean), 13.6 cm^3^ (5.6, 20.5 cm^3^) (median, IQR), (0.9—93.3 cm^3^) (range)	Ablation volume and extent of ablation	11.1 cm^3^ (mean), 9.9 cm^3^ (5.2, 16.4) (median, IQR), 1.0—28.1 cm^3^ (range)//75.8 ± 29.6% (mean), 91.9% (29.6%, 100.0%) (median, IQR)
Schroeder (2014)	Retrospective case series	6	44.1 cm^3^, 9.3 cm^3^, 7.2 cm^3^, 6.3 cm^3^, 5.3 cm^3^, 5.8 cm^3^, 27.8 cm^3^, 7.5 cm^3^	TDT coverage yellow and blue line	Yellow: 92% (77.7—100%), blue: 82.4% (42.7—100%) (means, ranges)
Sloan (2013)	Prospective case series	10	6.8 ± 5 cm^3^ (2.6—19 cm^3^) (mean, range)*	Volume and percentage of tumor treated at intended dose (VTT and PTT) and volume of tumor outside intended dosage line	VTT: 4.99 ± 3.24 cm^3^ (mean, SD)//PTT: 78 ± 12% (means, SDs) Volume of tumor outside intended dosage line: 2.04 ± 2.14 cm^3^ (mean, SD)*
Tovar-Spinoza (2016)	Retrospective case series	11	6.79 cm^3^ (mean)*	Immediate post ablation volume	IPA: 7.86 cm^3^ (mean)//1-3 m: 6.5 cm^3^, 4-6 m: 2.55 cm^3^, 7-9 m: 3.25 cm^3^, 10-12 m: 3 cm^3^ and 15-36 m: 1.1 cm^3^ (averages) (estimates from figure!)*
Traylor (2019)	Retrospective case series	27	N.R	Percentage of total ablation and residual tumor volume in incomplete ablation	Total ablation: 51.5%. In remaining 48.5%: residual tumor volume: 0.50 cm^3^ (0.03—4.61 cm^3^) (median, range)
Traylor (2019)	Retrospective case series	13	4.66 cm^3^ (median)*	Postoperative ablation cavity volume	IPA: 6.29 cm^3^ (median)//1moPO: 4.42 cm^3^, 3moPO: 4.16 cm^3^, 6moPO: 2.63 cm^3^, 9moPO: 2.90cm^3^, 12moPO: 3.11 cm^3^ (medians)*
Xue (2023)	Prospective case series	18	3.01 cm^3^ (0.40—7.40 cm^3^) (average, range)*	Complete ablation rate (IPA) and increase in volumetric size (on 30-day MRI)	92.4% (29.2—100%) (average, range)//2.28-fold (1.21—4.88-fold) (average, range)*

Abbreviations: *N.R. *not reported, *SD *standard deviation, *SE *standard error, *IQR *interquartile range, *CI *confidence interval. An asterisk (*) denotes that individual patient measurements were also reported in the respective paper

**Table 2:** Study characteristics and outcomes for studies focusing on epilepsy indications

Author	Study design	Sample size	Pre-operative/target volume or area	Definition of ablated volume	Post-LITT measurement of ablated volume
Alexander (2019)	Retrospective case series	4	5.89 ± 1.53 cm^3^ (mean, SD)*	Ablation volume	3.20 ± 1.92 cm^3^ (mean, SD)*
Aung (2023)	Retrospective case series	16	N.R	Ablation volume	2.59 cm^3^ (mean), 2.48 cm^3^ (0.93—6.7 cm^3^) (median, range)
Donos (2018)	Retrospective case series	43	N.R	Percentage of ablation	Amygdala: 73.7 ± 13.4%, hippocampus: 70.9 ± 12.6%, para hippocampal gyrus: 30.8 ± 9.9%, entorhinal cortex: 28.3 ± 15.3% (medians, median absolute deviations)
Gadgil (2019)	Retrospective case series	58	0.52 cm^3^ (0.06—14.49 cm^3^) (median, range)	Volume of ablated tissue and postoperative residual volume and percentage	ceT1: 0.33 cm^3^ (0.01—5.01 cm^3^); DWI: 0.45 cm^3^ (0—3.87 cm^3^); + 3 months: 0.15 cm^3^ (0.01—3.94 cm^3^) (medians, ranges) Residual: 50% (0—95.8%) (medians, ranges)
Grewal (2019)	Retrospective case series	23	5100 mm^3^ (mean), 4900 mm^3^ (median) (estimate from figure)*	Ablation volume and percentage hippocampal/amygdala ablation	6888 mm^3^ (3045.41–11,560.64 mm^3^)//Hippocampus: 65% (28–85%); amygdala: 43% (1%–80%) (medians, ranges)*
Gupta (2020)	Retrospective case series	35	N.R	Ablation volume	8.84 ± 7.54 cm^3^ (mean, SD)
Hwang (2022)	Retrospective case series	44	Hippocampus: 3.71 cm^3^ (3.06—4.19 cm^3^)//Amygdala: 1.70 cm^3^ (1.51—2.04 cm^3^) (medians, IQRs)	Ablation volume	4.27 cm^3^ (2.92–5.89 cm^3^) (median, IQR)
Hwang (2022)	Retrospective case series	39	Amygdala: 1.8 cm^3^ (1.5—2.2 cm^3^)//Hippocampus: 3.8 cm^3^ (3.4—4.5 cm^3^)//Piriform cortex 0.41 cm^3^ (0.36—0.46 cm^3^) (medians, IQRs)	Ablation volume and ablation percentage	4.95 cm^3^ (3.04–6.17 cm^3^)//Amygdala: 40.7% (26.3–51.3%); Hippocampus: 42.9% (26.3–53.1%); Piriform cortex 7.9% (0.4–18.8%) (means, IQRs)
Ibrahim (2018)	Retrospective case series	17	N.R	Ablation volume	Seizure-free: 5142 mm^3^; not seizure-free 5114 mm^3^
Infante (2024)	Retrospective case series	28	NA, 286.52 cm^3^, NA, 277.67 cm^3^, 1945.90 cm^3^, 1255.97 cm^3^, 1804.39 cm^3^, 2872.51 cm^3^, 3036.45 cm^3^, 145.65 cm^3^, 58.01 cm^3^, NA, 437.32 cm^3^, 3252.52 cm^3^, 503.35 cm^3^, 2566.14 cm^3^, 2963.24 cm^3^, 3109.29 cm^3^, 1182.12 cm^3^, 4049.38 cm^3^, 3243.86 cm^3^, 4771.63 cm^3^, 3355 cm^3^, 106 cm^3^, 1591 cm^3^, 3061 cm^3^, 4820.5 cm^3^, 2774.98 cm^3^, 360 cm^3^, 152 cm^3^, 2345.96 cm^3^, 162 cm^3^	Ablation volume and planned volume coverage	3060.55 mm^3^ (mean)//64.34% *
Jermakowicz (2018)	Retrospective case series	30	N.R	Ablation volume and TDEs	IPA MRI: 5478 ± 1195 cm^3^//+ 6 months MRI: 2440 ± 759 cm^3^//IPA MRI coronal: 565 ± 97 mm^2^//IPA MRI sagittal: 479 ± 103 mm^2^//TDE axial: 493 ± 81 mm^2^//TDE sagittal: 384 ± 62mm^2^
Jermakowicz (2017)	Retrospective case series	23	N.R	Total ablated volume (TAV) and percentages of ablation	Engel I: 3160 ± 190 mm^3^ (TAV), hippocampus: 80 ± 2%, amygdala: 54 ± 6%//Engel II-IV: 2880 ± 150 mm^3^ (TAV), hippocampus: 75 ± 7%, amygdala: 90 ± 5% (means ± SE)
Kang (2016)	Retrospective case series	20	N.R	Ablated volume	Class I: 5.4 cm^3^, Class II/III/IV: 3.2 cm^3^ (median)
Kim (2021)	Retrospective case series	35	Amygdala: ILAE Class I-II: 1.70 ± 0.07 cm^3^; Class III-VI: 1.83 ± 0.10 cm^3^//Hippocampus: Class I-II: 3.81 ± 0.20 cm^3^; Class III-VI: 4.16 ± 0.18 cm^3^	Total ablation volume and hippocampus/amygdala ablated	TAV: LAE Class I-II: 4.24 ± 0.47 cm^3^; Class III-VI: 3.37 ± 0.34 cm^3^//Amygdala: Class I-II: 25.17 ± 7.63%; Class III-VI: 18.99 ± 4.34%//Hippocampus: Class I-II: 30.62 ± 5.18%; Class III-VI: 19.61 ± 4.57%
Kim (2023)	Retrospective case series	27	Amygdala: ILAE Class I: 1.84 ± 0.08 cm^3^; Class II-VI: 1.91 ± 0.12 cm^3^//Hippocampus: Class I: 4.13 ± 0.22 cm^3^; Class II-VI: 4.35 ± 0.26 cm^3^ (means ± SEMs)	Total volume ablated	Amygdala: ILAE Class I: 44.73 ± 7.00%; Class II-VI: 33.45 ± 4.16%//Hippocampus: Class I: 47.40 ± 3.58%; Class II-VI: 36.18 ± 4.52% (means, SEMs)
Kim (2022)	Retrospective case series	33	PC: ILAE Class I: 0.37 ± 0.03 cm^3^; Class II-VI 0.36 ± 0.03 cm^3^//Amygdala: Class I: 1.84 ± 0.08 cm^3^; Class II-VI: 1.91 ± 0.12 cm^3^//Hippocampus: Class I: 4.13 ± 0.22 cm^3^; Class II-VI: 4.35 ± 0.26 cm^3^	Total ablation volume and PC/amygdala/hippocampus ablated	TAV: ILAE Class I: 5.33 ± 0.41 cm^3^; ILAE Class II-IV: 4.28 ± 0.35 cm^3^//PC: Class I: 10.16 ± 3.11%; Class II-VI: 3.30 ± 1.14%//Amygdala: Class I: 29.88 ± 8.14%; Class II-VI: 31.82 ± 4.46%//Hippocampus: Class I: 37.48 ± 6.81%; Class II-VI: 33.08 ± 4.54%
Ko (2022)	Retrospective case series	24	N.R	Total ablation volume (TAV)	SF: 6767 mm^3^ (4950—11.144 mm^3^); NSF 5763 mm^3^ (3077—8607 mm^3^) (medians, ranges)
Lombardi (2023)	Retrospective case series	20	241.4 ± 168.5 mm^2^ (mean ± SD)	Percentage of intraoperative ablation compared to initial HH size	Intra-op T2: 101.4 ± 37.7%//Intra-op ceT1: 93.4 ± 29.2%//Intra-op DWI excluding rim: 31.0 ± 11.9%//Intra-op DWI including rim: 87.2 ± 34.8% Residual: 175.8 ± 133.6 mm^2^ (mean, SD)
Malcolm (2021)	Retrospective case series	4	0.6 cm^3^, 2.6 cm^3^, 4.2 cm^3^, 0.92 cm^3^	Ablation volume, resulting lesion size and relative volume reduction	3.8 cm^3^, 1.1 cm^3^, 8.2 cm^3^, 1.0 cm^3^ Lesion size volume reduction: 0.3 cm^3^, 0.7 cm^3^, 1.2 cm^3^, 0.74 cm^3^//Relative volume reduction: −50%, −73%, −72%, −20%
McCracken (2016)	Prospective case series	5	0.36 cm^3^, 0.43 cm^3^, 0.22 cm^3^, 2.54 cm^3^, 0.79 cm^3^	Ablation volume and percent lesion ablated	0.91 cm^3^, 4.23 cm^3^, 1.89 cm^3^, 4.17 cm^3^, 4.07 cm^3^//80%, 90%, 94%, 95%, 98% (mean 91.4%)
Mithani (2021)	Retrospective case series	4	773.13 mm^3^, 432.18 mm^3^, 239.32 mm^3^, 1253.24 mm^3^	Volume of ablation	707.66 mm^3^, 406.25 mm^3^, 218.80 mm^3^, 1097.58 mm^3^
Morris (2017)	Retrospective case series	13	N.R., 5.690 cm^3^, N.R., N.R., N.R., 0.521 cm^3^, 0.976 cm^3^, N.R., N.R., N.R., N.R., N.R., N.R. (Mesial temporal sclerosis all N.R.)	Intraoperative, postoperative and residual enhancement volumes	IPA: 9.279 cm^3^, 8.105 cm^3^, 10.499 cm^3^, 9.242 cm^3^, 7.948 cm^3^, 2.624 cm^3^, 0.865 cm^3^, 7.925 cm^3^, 9.234 cm^3^, 8.883 cm^3^, 9.130 cm^3^, 7.497 cm^3^, 6.396 cm^3^//± 6 m post-op: 2.246 cm^3^, 1.2076 cm^3^, 2.775 cm^3^, 0.870 cm^3^, 2.836 cm^3^, 0.290 cm^3^, no enhancement, 0.889 cm^3^, 3.472 cm^3^, 3.870 cm^3^, 3.298 cm^3^, 4.348 cm^3^, 4.278 cm^3^ Residual enhancement + 6 months: 24.21%, 11.77%, 26.44%, 9.41%, 35.68%, 11.07%, N.A., 11.21%, 37.60%, 43.57%, 36.12%, 57.99%, 66.89%
Ordaz (2023)	Retrospective cohort	13 (of which 9 LITT)	N.R	Percentage of CC ablation	74.2%
Satzer (2020)	Retrospective case series	6	0.7 cm^3^ (0.2—3.2 cm^3^) (median, range) (T2-hypointense hemosiderin ring)*	Ablation volume	IPA contrast-enhancing region: 2.3 cm^3^ (0.5—4.6 cm^3^) (median, range)//6 m post-op T2-hypointense lesion volume: 0.5 cm^3^ (0.1—2.9 cm^3^) (median, range)*
Satzer (2021)	Retrospective case series	28	N.R	Ablation volume	Seizure freedom: 4.4 ± 2.0 cm^3^ (mean, SD), persistent seizures: 2.7 ± 1.1 cm^3^ (mean, SD) (first LITT)
Shofty (2021)	Retrospective case series	17	N.R	Ablation volume	4.7 mm^3^, 2.7 mm^3^, 4.4 mm^3^, 2.7 mm^3^, 3.9 mm^3^, 6.4 mm^3^, 4.9 mm^3^, 0.3 mm^3^, 2.7 mm^3^, 6.1 mm^3^, 0.2 mm^3^, 2.6 mm^3^, 6.78 mm^3^, 5.5 mm^3^, 2.5 mm^3^, 3.4 mm^3^
Slingerland (2023)	Retrospective cohort	74 (of which 27 LITT)	N.R	Ablation treated tissue volume	8.8 ± 5.02 cm^3^ (mean, SD)*
Tao (2020)	Retrospective case series	10	N.R	Percentage of CC ablated	53 ± 7% (mean, SD)
Tao (2018)	Prospective case series	21	N.R	Ablation volume and percentage ablated amygdahippocampal complex	1.5 cm^3^, 0.6 cm^3^, 1.8 cm^3^, 2.7 cm^3^, 5.3 cm^3^, 2.1 cm^3^, 1.0 cm^3^, 1.9 cm^3^, 2.8 cm^3^, 0.6 cm^3^, 2.0 cm^3^, 1.9 cm^3^, 2.2 cm^3^, 1.9 cm^3^, 1.9 cm^3^, 1.2 cm^3^, 2.4 cm^3^, 5.9 cm^3^, 6.6 cm^3^, 1.8 cm^3^, 2.8 cm^3^//44%, 17%, 47%, 73%, 92%, 45%, 26%, 54%, 87%, 19%, 69%, 51%, 58%, 51%, 73%, 87%, 92%, 100%, 100%, 96%, 90%
Willie (2014)	Prospective case series	13	N.R	Total ablation volume	5.3 ± 1.1 cm^3^ (mean, SD), 5.32 cm^3^ (3.52—7.59) (median, range)//Hippocampus: 63 ± 10% (mean, SD), 62.5% (54—86%) (median, range)//Amygdala: 54 ± 27% (mean, SD), 57.5% (4—88%) (median, range)//AHC: 60 ± 9.7% (mean, SD), 59.5% (46—76%) (median, range)
Willie (2019)	Retrospective case series	19	0.7 ± 0.6 cm^3^ (0.1—2.5 cm^3^) (mean, range)*	Ablation zone, absolute and relative change lesion volume	3.8 ± 2.6 cm^3^ (0.6—10.0 cm^3^) (mean, range) Volume at FU: 0.2 ± 0.2cm^3^ (mean)//Relative change: −90%, −80%, −100%, −91%, −50%, −67%, NA, NA, −50%, −100%, −89%, −92%, −86%, −44%, NA, −20%, −60%, NA, NA
Wu (2015)	Retrospective case series	19	Amygdala: 1.27 cm^3^, 1.31 cm^3^, 1.47 cm^3^, 1.91 cm^3^, 1.77 cm^3^, 1.49 cm^3^, 1.28 cm^3^, 1.52 cm^3^, 1.80 cm^3^, 1.81 cm^3^, 1.64 cm^3^//Hippocampus: 3.58 cm^3^, 2.64 cm^3^, 3.97 cm^3^, 3.61 cm^3^, 3.17 cm^3^, 4.47 cm^3^, 3.00 cm^3^, 3.50 cm^3^, 3.54 cm^3^, 2.86 cm^3^, 3.05 cm^3^	Volume of ablation and coverage	6.48 cm^3^ (average)//Hippocampus: 56%, amygdala: 52% (averages)*
Zheng (2023)	Retrospective case series	5	N.R	Residual volumes and residual percentages	MTL: 10,806 mm^3^, 10,954 mm^3^, 7892 mm^3^, 12,095 mm^3^, 11,252 mm^3^, amygdala: 872 mm^3^, 1522 mm^3^, 415 mm^3^, 3386 mm^3^, 4465 mm^3^//MTL: 75.2, 76.3%, 55.0%, 78.7%, 73.2%, amygdala: 76%, 37%, 36.2%, 59.7%, 90.4%. (First LITT)

Abbreviations: *N.R. *not reported, *SD *standard deviation, *SE *standard error, *IQR *interquartile range, *CI *confidence interval. An asterisk (*) denotes that individual patient measurements were also reported in the respective paper

**Table 3:** Study characteristics and outcomes for studies focusing on oncology and epilepsy (and other) indications

Author	Study design	Sample size	Pre-operative/target volume or area	Definition of ablated volume	Post-LITT measurement of ablated volume
Attaar (2015)	Retrospective case series	72	Tumors: 10.9 ± 17.3 cm^3^ (average) (Other etiologies N.R.)*	Percentage of ablation	74% total ablation, 26% subtotal (< 90%) ablation
Dadey (2016)	Retrospective case series	5	17.0 cm^3^, 5.4 cm^3^, 8.7 cm^3^ (2 × N.R.)	Yellow/blue TDT volume and percentage treated to yellow/blue	Yellow: 31.1 cm^3^, 18.4 cm^3^, 12.0 cm^3^, 14.4 cm^3^, 23.8 cm^3^, blue: 24.2 cm^3^, 13.6 cm^3^, 8.3 cm^3^, 10.3 cm^3^, 19.4 cm^3^//Yellow: 100%, 75.5%, 100%, 86.6%, 100%, blue: 100%, 62.2%, 100%, 79.2%, 100%
Jensdottir (2023)	Retrospective case series	30	1.2 cm^3^ (0.5—2.9 cm^3^) (median, IQR)	Lesion coverage	100% (90—100%) (median, IQR)
Lauren (2018)	Retrospective case series	10	6.94 ± 5.03 cm^3^ (mean ± SD)*	Treated volume and ablation percentage	9.58 ± 6.68 cm^3^, 263 ± 436% (means, SD)*
Liang (2021)	Retrospective case series	20	N.R	TDE volume	Method A: 3.44 ± 1.96 cm^3^, Method B: 4.83 ± 1.53 cm^3^ (averages)*

Abbreviations: *N.R. *not reported, *SD *standard deviation, *SE *standard error, *IQR *interquartile range. An asterisk (*) denotes that individual patient measurements were also reported in the respective paper

The risk of bias assessment indicated considerable variability in the methodological quality of the included studies. Most studies demonstrated adequate selection criteria and outcome assessment but lacked comprehensive adjustment for confounding factors. Of the 77 studies assessed, 74 had a moderate risk of bias (score 4–6), while 3 studies had a low risk of bias (score 7). Studies with higher scores generally accounted for key risk factors, while lower-scoring studies lacked sufficient methodological rigor. The full results are available in Supplementary Item 3.

### Reporting on pre- and post-ablation volumes

Various terms were used to describe the measured post-LITT ablation zone, with "extent of ablation" and "ablation volume" being the most frequently cited throughout the papers. The outcomes were reported as volumes or percentages and as medians or means. In some papers, the type of central tendency measure and/or measure of variability was not reported. Twenty-four studies (31%) Did not report pre-operative volume or target area, with this being most common in epilepsy studies, where 18 studies lacked information on pre-operative volumes or target areas. In 5 (%) oncology studies and 1 (%) study with both epilepsy and oncology indications, this pre-operative measurement was not reported. Pre-operative measurements were reported as either volumes or area and as medians or means. The results concerning pre-operative/target volume, description of post-LITT measurement, and outcomes are summarized in Tables 1, 2 and, 3.

### Methods used for measurement of ablation volume or percentage after LITT

In 65 studies (84%), post-LITT volumes were delineated based on anatomical imaging using conventional MR imaging, while 10 studies (13%) assessed thermal damage.

**Fig. 2:**
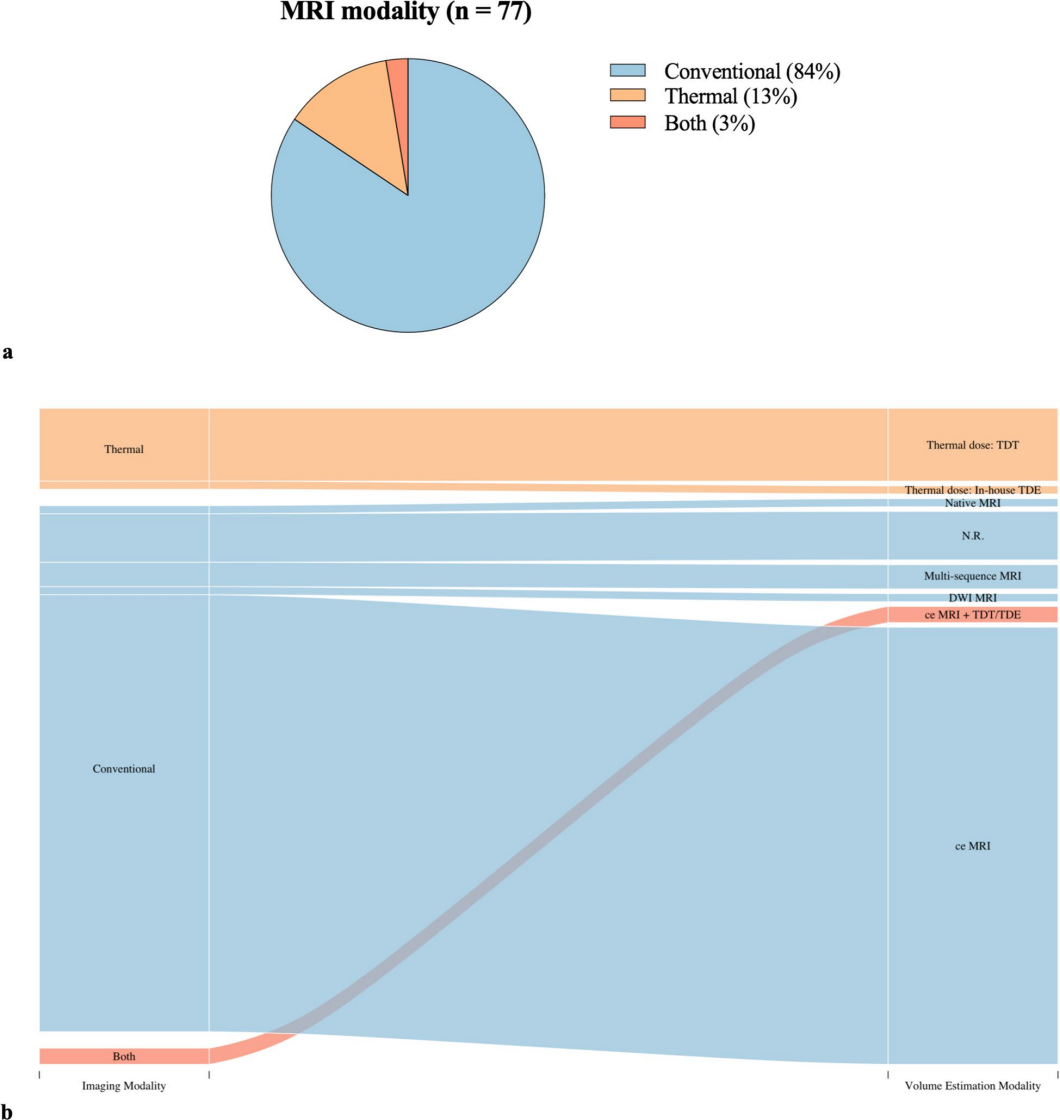
Imaging modalities (**2a**) and volume estimation modalities applied for first volume assessment (**2b**) after LITT. Abbreviations: N.R. = not reported, TDT = Thermal Dose Threshold, TDE = Thermal Damage Estimate, DWI = diffusion-weighted imaging, ce = contrast-enhanced

Among the studies utilizing conventional MRI sequences, contrast-enhanced imaging, including contrast-enhanced T1, T1 dynamic contrast-enhanced (DCE), and T1 magnetization-prepared rapid acquisition gradient echo (MPRAGE) sequences, was used to delineate the ablation zone in 55 studies (71%). Other studies relying on conventional MRI utilized diffusion-weighted imaging (DWI) or non-contrast native sequences such as T1-weighted imaging. Five studies reported the use of MRI but did not specify the sequences applied. All ten studies employing thermal measurement methods utilized a thermal dose model: nine of these used thermal damage threshold (TDT) lines. In these cases, the TDT lines were overlaid on the pre-LITT imaging to determine the residual tumor volume, which was then subtracted from or divided by the pre-treatment tumor volume to estimate the percentage of ablated tissue. One study implemented an in-house developed thermal damage model. The two studies that applied both conventional and thermal methods combined contrast-enhanced imaging with a thermal dose model (either TDT lines or the thermal damage estimate (TDE)). In the study using TDE-based analysis, the postoperative ablation zone was quantified by measuring the Distance from the laser catheter to the ablation margin at 20 equidistant points along the catheter axis, perpendicular to its trajectory. These measurements were then used to estimate the ablation area. The methods used for volume determination are visualized in Fig. 2. In some studies, different volume estimation modalities were employed for the initial and follow-up measurements. To preserve clarity, Fig. 2b Displays only the modalities used for the initial measurement. Detailed results are provided in Supplementary Item 4.

**Fig. 3:**
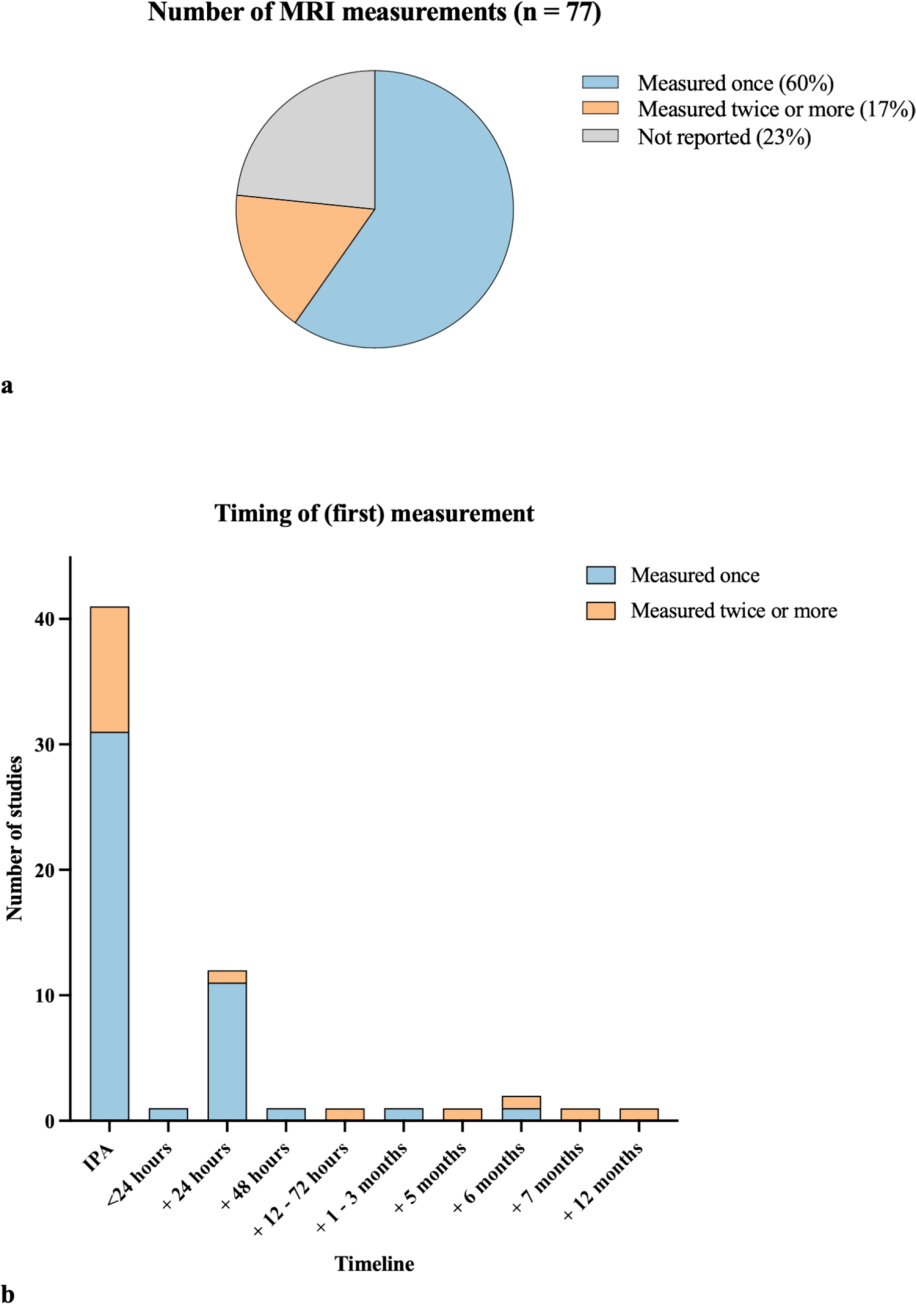
Number of volume measurements **(3a)** and timing of first measurement **(3b)**

In 46 studies (60%), the ablation volume was calculated once following treatment, either immediately after LITT or later. Thirteen studies (17%) measured the ablation volume two or more times; all measured immediately after treatment and then later during follow-up. Thirty-nine studies (51%) assessed the ablation volume intra-operatively, thus immediately post-ablation (IPA). One study performed the first measurement within 24 h post-operatively, twelve studies measured it 24 h post-operatively, one study measured it 48 h post-operatively, one study measured it between 12 and 72 h post-operatively, two studies measured it 1 to 3 months post-operatively, and one study measured it 6 months post-operatively. Eighteen studies (23%) did not report the timing of the ablation volume measurements. No trends were observed regarding the frequent use of specific methods in papers focused on epilepsy or oncology. The number of ablation volume measurements and the timing of these measurements are visualized in Fig. 3. Given the substantial heterogeneity in follow-up time points among studies that performed two or more measurements, only the time points of the initial measurements were included in Fig. 3b to maintain clarity. Detailed results regarding the timing of measurements are provided in supplementary Item 4.

Among the 60 studies that employed contrast-enhanced sequences, 30 studies (50%) explicitly reported whether the enhancing rim was included in the volumetric assessment of the ablation zone. In 25 oncology studies the rim was included in their measurements, and in 5 the rim was excluded from their measurements. Three studies provided inconclusive descriptions regarding rim inclusion, and 27 studies did not report any information on rim handling. Apart from the studies using contrast-enhanced sequences, one study assessing DWI evaluated volumes both with and without the inclusion of the enhancing rim. Additionally, one study that did not explicitly specify the imaging sequences used did provide details on rim handling; the enhancing rim was excluded from their volumetric measurements.

**Fig. 4:**
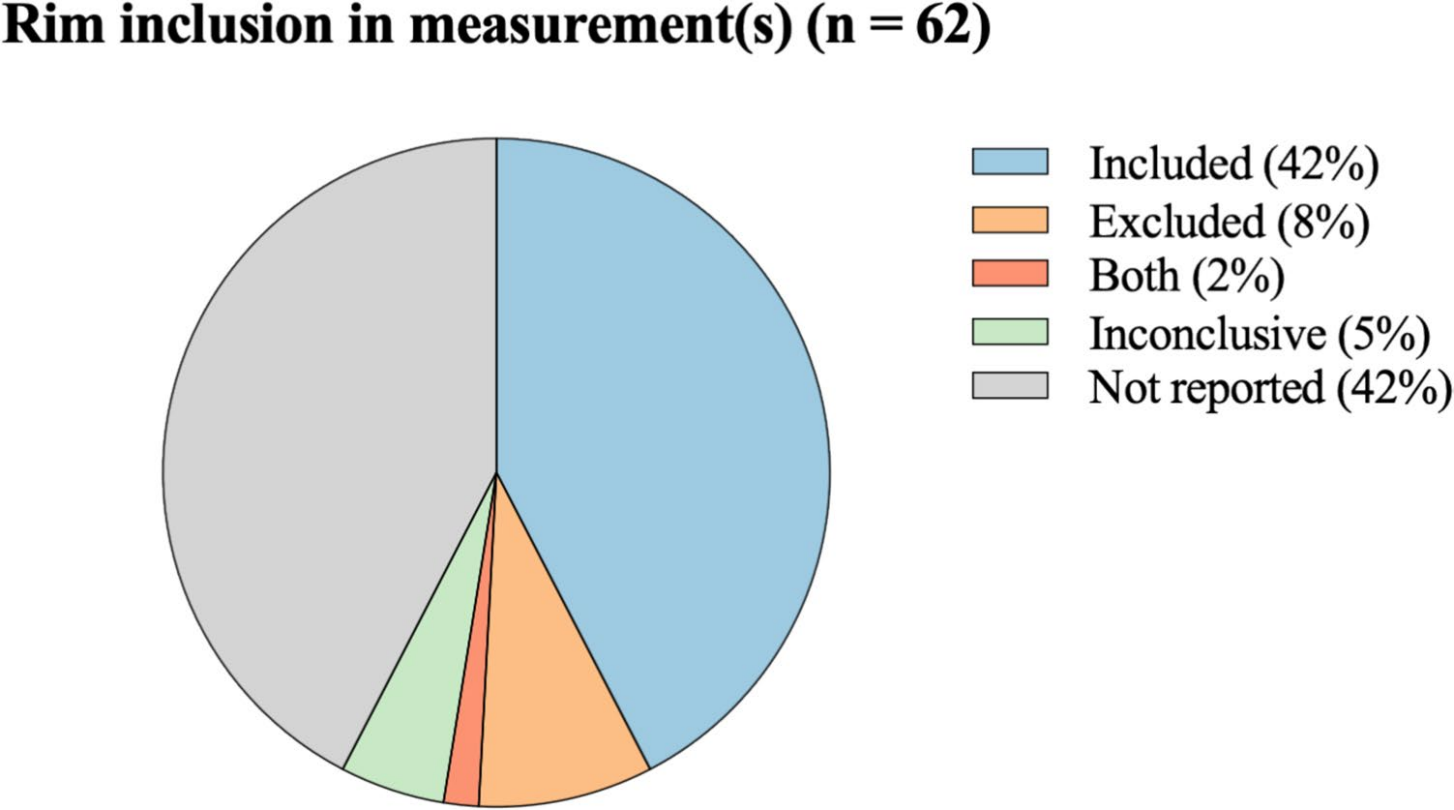
Hyperenhancing rim in- or exclusion from ablation volume measurements

Among the oncology papers, 16 studies addressed the inclusion or exclusion of the rim: ten studies included the rim, four excluded it, two provided inconclusive descriptions, and twelve provided no information. In the epilepsy papers, 16 studies reported their method of measurement. Of these, 14 included the rim in their measurements, one study conducted measurements both with and without the rim (DWI) and one study provided an inconclusive description. Among the papers investigating both oncology and epilepsy indications (as well as other indications), 3 described their methods; one included the rim, while two excluded it from their measurements. Data on rim in- or exclusion from measurements is visualized in Fig. 4. This figure includes only data from studies that utilized contrast-enhanced imaging or explicitly reported on rim inclusion. Studies employing non-contrast-enhanced imaging or thermal dose calculations were excluded, as no enhancing rim is present in these modalities.

All data on the methods used to measure ablation volume after LITT are available as Supplementary Item 4.

## Discussion

This systematic review of 77 studies on laser interstitial thermal therapy (LITT) in intracranial lesions provides a comprehensive and up to date overview of methods used for ablation zone definition and volumetry in LITT for neurosurgical applications. The results show that the majority of studies relied on ce-T1 MRI for volumetric assessment, though thermal MRI was occasionally utilized. Notably, 46 studies performed a single volume measurement immediately after treatment, whereas others conducted assessments at later time points. This is of importance as the timing of measurement is known to significantly influence the measured volume outcomes [10, 69]. Of 60 studies that have used contrast-enhanced MRI for ablation zone assessment, only 50% of studies explicitly stated whether the enhancing rim was included in their calculations. We also found high variability in ablation zone measurement methodology and underreporting of related critical parameters.

Another important finding of this review is the variability in terminology and outcome measures used to define ablation extent or volume. Terms like "ablation volume," "percentage of ablation," "increase in volumetric size," and "postoperative ablation cavity volume" are inconsistently defined and reported. This lack of standardization complicates result interpretation and cross-study comparisons, highlighting the need for a unified nomenclature and measurement framework.

The observed heterogeneity in ablation volume measurement aligns with findings from prior studies, which have also reported inconsistencies in imaging modalities, segmentation methods, and measurement time points. Studies such as those by Haskell-Mendoza et al. have specifically highlighted the lack of consensus regarding the inclusion of the enhancing rim in volumetric calculations [29]. Only one study included in this review directly compared measurements with and without the enhancing rim [48]. They found that the post-operative enhancing rim appears to overestimate the extent of the final irreversible damage. Based on the existing literature, there is insufficient evidence to determine which method is superior. Therefore, it is not possible to make a well-founded statement on this matter. It is important that future studies clearly describe their methodologies and that further research is conducted on the significance of the enhancing rim following LITT, allowing for the implementation of a standardized approach in the future.

Furthermore, previous research has shown that variations in the timing of post-ablation imaging can significantly affect the measured ablation volumes due to tissue dynamics and healing responses [10, 69]. In this systematic review, thirteen studies assessed post-LITT volumes at different time points [3, 10, 38, 50, 51, 55, 60, 69, 73, 77, 87, 90, 92]. Their findings reveal that post-LITT volume initially increases immediately after the procedure, likely due to inflammatory responses and thermal necrosis [74], before gradually decreasing over time. This suggests that the timing of post-operative zone measurement plays a critical role in determining the final measured volume.

We encourage future studies to clearly define and report the ablation volume as the three-dimensional region of tissue treated by the laser, characterized by irreversible thermal damage, or the extent of ablation, described as the proportion of definitively damaged tissue relative to the preoperative target volume. When reporting the extent of ablation, it is important to overlay the area of definitive thermal damage with the preoperative target area, with the extent defined by the degree of overlap. We suggest that ablation volume should be measured using contrast-enhanced T1-weighted MRI at least once, immediately following the procedure. We recommend measuring ablation volume on the immediate post-ablation MRI, as this timepoint is most consistently used across studies and minimizes measurement variability. It is also routinely acquired in clinical practice, avoiding the need for additional imaging. While this scan is often assessed visually in clinical practice, standardized quantitative measurements could improve treatment evaluation and clinical decision-making and may enable real-time ablation adjustments in the future. In the case that additional imaging is desired for the purpose of ablation volume assessment, it may be considered to perform it at 24 h after the procedure [10, 69]. If alternative measurement methods or definitions are used, these should be clearly explained to ensure clarity regarding what is measured and by which imaging techniques. For example, three-dimensional thermal damage estimates may be determined as complementary measurement. In addition, we recommend that at minimum the following parameters be reported: timing of measurement(s), imaging modalities used (e.g., contrast-enhanced T1, DWI, MR thermography, TDT lines), segmentation methods (manual or (semi-)automated), and whether the enhancing rim was included or excluded from the measurements. Standardized reporting of these parameters will facilitate better interpretation and comparison of results across studies and represents the first step toward establishing a standardized method for measuring post-LITT ablation volumes.

A key strength of this systematic review is its comprehensive assessment of the existing literature, systematically identifying inconsistencies in imaging-based ablation zone assessment. This review identifies areas of underreporting and methodological variability, offering a foundation for enhancing future research practices through four recommendations (Table 4). However, several limitations should also be discussed. First, the heterogeneity of study designs and methodologies limited direct comparisons across studies. Besides this, the majority of the included studies were retrospective in nature and exhibited a moderate risk of bias. Second, no studies have been identified that directly compare different methodologies for measuring post-LITT volumes. A limited number of studies have validated the EOA in relation to clinical outcomes, such as survival, but often lack detailed information on methods used to define the EOA [20, 40, 67].

**Table 4:** Recommendations for the reporting of methods used for post-LITT volume assessment

Recommendations	
Pre-operative volume	Determine the pre-operative target volume prior to the LITT procedure
Definition of ablation volume	Report the measured outcome as the ablation volume, defined as the intersection of the pre-operative volume and the ablated volume and expressed in cubic centimeters (cc), cubic millimeters (mm^3^), or cubic centimeters (cm^3^), or alternatively, report the extent of ablation (EOA) as a percentage of the intersection between the pre-operative volume and the ablated volume
Modality and timing	Ablation volumes should be measured using contrast-enhanced T1-weighted MRI sequences. Measurements should be done at least once immediately following the LITT procedure
In- or exclusion of enhancing rim	Clearly specify whether the enhancing rim was included in or excluded from the measurements

The variability in ablation zone measurement has significant implications for clinical decision-making and treatment evaluation. Inconsistent reporting and methodological heterogeneity hinder the ability to establish reliable response criteria, complicating patient selection, post-procedural monitoring, and long-term outcome assessments. The lack of standardized imaging protocols also impacts the reproducibility of results, limiting the generalizability and comparability of findings across institutions. Establishing uniform guidelines for ablation zone measurement is crucial to ensuring accurate interpretation of treatment efficacy and facilitating advancements in LITT application.

## Conclusion

This systematic review offers a comprehensive overview of the employed methods for defining and measuring the post-LITT ablation zone. The most commonly utilized technique is contrast-enhanced T1-weighted MRI, typically performed once immediately after LITT. However, reporting on various aspects of the methods used to measure ablation volume remains limited and heterogeneous. To improve result interpretation, enhance study comparability, increase clinical applicability, and ultimately achieve better patient outcomes, standardized reporting and methodological consistency are essential. To this end, we propose four recommendations that provide a framework for achieving greater standardization.

## Supplementary Information

Below is the link to the electronic supplementary material.

ESM 1 (PDF 127 KB)

ESM 2 (PDF 77.3 KB)

ESM 3 (PDF 184 KB)

ESM 4 (PDF 188 KB)

## Data Availability

The full data extraction table generated and analyzed during this study is not included in this paper due to its format and size but is available from the corresponding author upon reasonable request.
