# Supplementary material for: Imaging-based techniques for ablation zone definition and volumetry after laser interstitial thermal therapy (LITT) for intracranial lesions: a systematic review
Source: Acta Neurochir (Wien). 2025 Oct 8;167(1):269. doi: 10.1007/s00701-025-06666-6 (PMC12507988; doi:10.1007/s00701-025-06666-6)
Supplement: Supplementary file 2 — (PDF 77.3 KB) [file 701_2025_6666_MOESM2_ESM.pdf]

Supplementary Material 2

Imaging-Based Techniques for Ablation Zone Definition and Volumetry after Laser Interstitial Thermal Therapy (LITT) for Intracranial Lesions: A Systematic Review

Céline L.G. Neutel, MD[1], Thomas M. Putinela[1], Maroeska M. Rovers, PhD[2], Pierre A. Robe, MD, PhD[3] Mark ter Laan, MD, PhD[1], Christiaan G. Overduin, PhD[2]

[1] Radboud university medical center, department of neurosurgery, Nijmegen, The Netherlands.

[2] Radboud university medical center, department of medical imaging, Nijmegen, The Netherlands.

[3] University medical center Utrecht, department of neurosurgery, Utrecht, The Netherlands.

Journal: Acta Neurochirurgica

Correspondence to: Céline Neutel, MD

Department of Neurosurgery, Radboud University Medical Center, Nijmegen, the Netherlands

Email: celine.neutel@radboudumc.nl

Supplementary item 2

Full Systematic Search

<u>Pubmed</u>

(“laser therapy”[mesh] OR "Laser interstitial thermal therapy"[tiab] OR "LITT"[tiab] OR “MRgLITT” [tiab] OR "SLA"[tiab] OR "Laser ablat*"[tiab] OR "Laser surg*"[tiab] OR "Laser Induced Thermal Therap*" [tiab] OR "laser interstitial therap*" [tiab] OR "laser thermal therap*" [tiab] OR "laser thermal ablat*" [tiab] OR "laser interstitial thermotherap*" [tiab] OR "interstitial laser thermotherap*" [tiab]) AND ("brain neoplasms"[mesh] OR "Neoplasms, Neuroepithelial" [mesh] OR “neuro-oncology”[tiab] OR "Intracranial lesion*"[tiab] OR “Neuroepithelial Neoplasm”[tiab] OR "Intracranial neoplasm*"[tiab] OR "Brain tumor*"[tiab] OR "Brain tumour*"[tiab] OR "glioma*"[tiab] OR "glial*"[tiab] OR "astrocytoma*"[tiab] OR "glioblastoma*"[tiab] OR "oligodendrogli*"[tiab] OR "oligoastrocyt*"[tiab] OR "GBM"[tiab] OR "epilepsy"[mesh] OR “epilep*”[tiab] OR "radiation necrosis"[tiab] OR "brain metastas*"[tiab] OR “cerebral metastas*”[tiab])

<u>Embase</u>

("Laser interstitial thermal therapy".ti,ab,kf. OR "LITT".ti,ab,kf. OR MRgLITT.ti,ab,kf. OR “SLA”.ti,ab,kf. OR “Laser ablat*”.ti,ab,kf. OR “Laser surg*”.ti,ab,kf. OR “Laser Induced Thermal Therap*" .ti,ab,kf. OR “laser interstitial therap*” .ti,ab,kf. OR “ laser thermal therap*” .ti,ab,kf. OR “laser thermal ablat*” .ti,ab,kf. OR “laser interstitial thermotherap*” .ti,ab,kf. OR “interstitial laser thermotherap*” .ti,ab,kf.) AND (exp brain tumor/ OR exp neuroepithelioma/ OR “neuro-oncology”.ti,ab,kf. OR "Intracranial lesion*".ti,ab,kf. OR “Neuroepithelial neoplasm”.ti,ab,kf. OR "Intracranial neoplasm*".ti,ab,kf. OR “Brain tumor*”.ti,ab,kf. OR “Brain tumour*”.ti,ab,kf. OR “glioma*”.ti,ab,kf. OR “glial*”.ti,ab,kf. OR “astrocytoma*”.ti,ab,kf. OR “glioblastoma*”.ti,ab,kf. OR “oligodendrogli*”.ti,ab,kf. OR “oligoastrocyt*”.ti,ab,kf. OR “GBM”.ti,ab,kf. OR exp epilepsy/ OR “epilep*”.ti,ab,kf. OR “radiation necrosis”.ti,ab,kf. OR “brain metastas*”.ti,ab,kf. OR “ cerebral metastas*”.ti,ab,kf.)
