# Supplementary material for: Imaging-based techniques for ablation zone definition and volumetry after laser interstitial thermal therapy (LITT) for intracranial lesions: a systematic review
Source: Acta Neurochir (Wien). 2025 Oct 8;167(1):269. doi: 10.1007/s00701-025-06666-6 (PMC12507988; doi:10.1007/s00701-025-06666-6)
Supplement: Supplementary file 3 — (PDF 184 KB) [file 701_2025_6666_MOESM3_ESM.pdf]

Supplementary Material 3

Imaging-Based Techniques for Ablation Zone Definition and Volumetry after Laser Interstitial Thermal Therapy (LITT) for Intracranial Lesions: A Systematic Review

Céline L.G. Neutel, MD[1], Thomas M. Putinela[1], Maroeska M. Rovers, PhD[2], Pierre A. Robe, MD, PhD[3] Mark ter Laan, MD, PhD[1], Christiaan G. Overduin, PhD[2]

[1] Radboud university medical center, department of neurosurgery, Nijmegen, The Netherlands.

[2] Radboud university medical center, department of medical imaging, Nijmegen, The Netherlands.

[3] University medical center Utrecht, department of neurosurgery, Utrecht, The Netherlands.

Journal: Acta Neurochirurgica

Correspondence to: Céline Neutel, MD

Department of Neurosurgery, Radboud University Medical Center, Nijmegen, the Netherlands

Email: celine.neutel@radboudumc.nl

Supplementary item 3

Full Risk of Bias assessment table

**Author**	**Year**	**Selection**				**Comparability**		**Outcomes**			**Total Quality Score**
		Representativeness of Exposed Cohort	Selection of Non-Exposed Cohort	Ascertainment of Exposure	Demonstration That Outcome of Interest Was Not Present at Start of Study	Adjust for the Most Important Risk Factors	Adjust for Other Risk Factors	Assessment of Outcome	Follow-Up Length	Loss to Follow-Up Rate	
Alexander	2019			*	*			*			3
Ashraf	2020	*		*	*			*	*	*	6
Attaar	2015			*	*			*			3
Aung	2023	*		*	*			*	*		5
Bartlett	2023	*		*	*			*	*		5
Bastos	2020	*		*	*	*	*	*		*	7
Beaumont	2018	*		*	*			*	*	*	6
Beechar	2018	*		*	*			*	*		5
Borghei-Razavi	2018	*		*	*			*	*		5
Carpentier	2012			*	*			*	*	*	5
Carpentier	2011	*		*	*			*	*	*	6
Chaunzwa	2017	*		*	*		*	*		*	6

Dadario	2022	*		*	*			*	*		5
Dadey	2016			*	*			*			3
Daggubati	2023	*	*	*	*			*			5
Donos	2018	*		*	*			*	*		5
Eichberg	2018			*	*			*		*	4
Gadgil	2019			*	*				*		3
Grewal	2019	*		*	*			*	*		5
Gupta	2020	*		*	*			*	*	*	6
Gurses	2024	*		*	*			*	*		5
Haskell-Mendoza	2024	*		*	*			*	*		5
Hwang	2022	*		*	*		*	*	*		6
Hwang	2022	*		*	*			*	*		5
Ibrahim	2018			*	*			*	*		4
Infante	2024	*		*	*			*	*		5
Jensdottir	2023			*	*			*	*		4
Jermakowicz	2018			*	*			*			3
Jermakowicz	2017			*	*		*	*	*		5
Kahn	1994	*		*	*			*	*		5
Kaisman-Elbaz	2023	*		*	*			*	*	*	6
Kang	2015	*		*	*			*	*		5
Kim	2021	*		*	*		*	*			5
Kim	2022			*	*		*	*	*		5
Kim	2022	*		*	*		*	*			5
Ko	2021			*	*			*	*		4
Laurent	2018	*		*	*			*	*		5
Liang	2020	*		*	*			*	*		5
Lombardi	2023	*		*	*			*	*		5

Luther	2021	*		*	*			*	*		5
Luther	2020	*		*	*			*	*		5
Malcolm	2021			*	*			*			3
Maraka	2018			*	*			*			3
McCracken	2016	*		*	*			*	*		5
Merenzon	2024	*		*	*			*			4
Merenzon	2023	*		*	*		*	*	*		6
Merenzon	2022	*	*	*	*			*	*		6
Missios	2013	*		*	*			*	*		5
Mithani	2021			*	*			*			3
Mohammadi	2014	*		*	*	*	*	*	*		7
Morris	2017			*	*			*	*		4
Muir	2022			*	*			*			3
Muir	2022	*		*	*			*			4
Murayi	2020	*		*	*			*	*		5
Ordaz	2023	*	*	*	*			*			5
Patel	2013			*	*			*			3
Patel	2015			*	*			*	*		4
Rammo	2018	*		*	*			*			4
Rao	2014	*		*	*			*	*	*	6
Reese	2024	*		*	*			*			4
Satzer	2020	*		*	*				*		4
Satzer	2021			*	*			*	*		4
Schroeder	2014	*		*	*			*			4
Shofty	2021	*		*	*			*	*		5
Slingerland	2023	*	*	*	*			*	*		6
Sloan	2013	*		*	*			*	*		5
Tao	2020			*	*			*	*		4
