# Supplementary material for: Imaging-based techniques for ablation zone definition and volumetry after laser interstitial thermal therapy (LITT) for intracranial lesions: a systematic review
Source: Acta Neurochir (Wien). 2025 Oct 8;167(1):269. doi: 10.1007/s00701-025-06666-6 (PMC12507988; doi:10.1007/s00701-025-06666-6)
Supplement: Supplementary file 4 — (PDF 188 KB) [file 701_2025_6666_MOESM4_ESM.pdf]

Supplementary Material 4

Imaging-Based Techniques for Ablation Zone Definition and Volumetry after Laser Interstitial Thermal Therapy (LITT) for Intracranial Lesions: A Systematic Review

Céline L.G. Neutel, MD[1], Thomas M. Putinela[1], Maroeska M. Rovers, PhD[2], Pierre A. Robe, MD, PhD[3] Mark ter Laan, MD, PhD[1], Christiaan G. Overduin, PhD[2]

[1] Radboud university medical center, department of neurosurgery, Nijmegen, The Netherlands.

[2] Radboud university medical center, department of medical imaging, Nijmegen, The Netherlands.

[3] University medical center Utrecht, department of neurosurgery, Utrecht, The Netherlands.

Journal: Acta Neurochirurgica

Correspondence to: Céline Neutel, MD

Department of Neurosurgery, Radboud University Medical Center, Nijmegen, the Netherlands

Email: celine.neutel@radboudumc.nl

Supplementary item 4

Table A: Study results for methods used for calculation of ablated volume or percentage of ablation for oncology papers

Author	Year	Imaging modality	Volume Estimation Modality	Timing	Software	In- or exclusion of enhancing rim
Ashraf	2020	Conventional MRI sequences	N.R.	+ 24 hours	OsiriX DICOM viewer	N.A.
Bartlett	2023	Conventional MRI sequences	T1 DCE	+24 hours	N.R.	N.R.
Bastos	2020	Conventional MRI sequences	ceT1	IPA	N.R.	N.R.
Beaumont	2018	Thermal MRI sequences	Thermal dose (TDT-line)	N.R.	NeuroBlate	N.A.
Beechar	2018	Conventional MRI sequences	ceT1 and T2 FLAIR	"Post-LITT", 0-90 days, 90-180 days, 180-270 days, 270-365 days	Iplannet	Including enhancing rim

Borghei-Razavi	2018	Thermal MRI sequences	Thermal dose (TDT-line)	N.R.	NeuroBlate and iPlan Brainlab	N.A.
Carpentier	2012	Conventional MRI sequences	ceT1	IPA	N.R.	N.R.
Carpentier	2011	Conventional MRI sequences	ceT1	N.R.	N.R.	Including enhancing rim
Chaunzwa	2017	Conventional MRI sequences	N.R.	IPA	Monteris M-vision software	N.A.
Dadario	2022	Conventional MRI sequences	N.R.	+24 hours	OsiriX	N.A.
Daggubati	2023	Conventional MRI sequences	ceT1 MPRAGE	N.R.	Horos	N.R.
Eichberg	2018	Conventional MRI sequences	ceT1	IPA	N.R.	N.R.
Gurses	2024	Conventional MRI sequences	ce	+24 hours	PACS	Including enhancing rim
Haskell-Mendoza	2024	Conventional MRI sequences	ceT1	+1-3 months	In-house developed deep-learning model	Including enhancing rim

Kahn	1994	Conventional MRI sequences	T1 + Gd-DTPA	+24 hours	N.R.	Including enhancing rim
Kaisman-Elbaz	2023	Thermal MRI sequences	Thermal dose (TDT-line)	IPA	iPlan software BrainLab	N.A.
Luther	2021	Conventional MRI sequences	ceT1	+24 hours + last follow-up	OsiriX	Including enhancing rim
Luther	2020	Conventional MRI sequences	ceT1	+24 hours	ImageJ	N.R.
Maraka	2018	Conventional MRI sequences	ceT1	+24 hours	BrainLab	N.R.
Merenzon	2024	Conventional MRI sequences	ceT1	+24 hours	PACS	Including enhancing rim
Merenzon	2023	Conventional MRI sequences	ceT1	+12–72 hours, +60–90 days, +120–180 days, and +210–365 days. Not specified which was used for	PACS	N.R.

				median calculation.		
Merenzon	2022	Conventional MRI sequences	ceT1	24 h post ablation	N.R.	Including enhancing rim
Missios	2014	Thermal MRI sequences	Thermal dose (TDT-line)	N.R.	Monteris M-vision software	N.A.
Mohammadi	2014	Thermal MRI sequences	Thermal dose (TDT-line)	Not specified	iPlan	N.A.
Muir	2022	Thermal MRI sequences	Thermal dose (TDT-line)	N.R.	iPlan software BrainLab	N.A.
Muir	2022	Conventional MRI sequences	ceT1	IPA	iPlan software BrainLab	*"The tumor margin was the ablation cavity"*
Murayi	2020	Conventional and thermal MRI sequences	ceT1 and Thermal dose (TDT-line)	N.R.	iPlan software	N.R.
Patel	2013	Conventional MRI sequences	ceT1	IPA, +24 hours and first follow-up	OsiriX	Including enhancing rim

Patel	2015	Conventional MRI sequences	ceT1	N.R.	OsiriX	Excluding enhancing rim
Rammo	2018	Conventional MRI sequences	ceT1	IPA, +2 weeks, +6 weeks, +2-3 months, +6 months, +1 year	BrainLab	Excluding enhancing rim
Rao	2014	Conventional MRI sequences	ceT1	+24 hours	N.R.	Including enhancing rim
Reese	2024	Conventional MRI sequences	ceT1	<24 hours	BrainLab	Excluding enhancing rim
Schroeder	2014	Thermal MRI sequences	Thermal dose (TDT line)	N.R.	N.R.	N.A.
Sloan	2013	Thermal MRI sequences	Thermal dose (TDT line)	+48 hours	N.R.	N.A.
Tovar-Spinoza	2016	Conventional MRI sequences	ceT1	IPA and every 3 months	N.R.	N.R.
Traylor	2019	Conventional MRI sequences	ceT1	N.R.	iPlan Software	N.R.

Traylor	2019	Conventional MRI sequences	ceT1	IPA, +1 month, +3 months, +6 months, +9 months, +12 months	iPlan software	*“The margin was the ablation cavity”*
Xue	2023	Conventional MRI sequences	ceT1	IPA, +30 days, +90 days, +180 days	3D Slicer	N.R.
Fadel	2022	Conventional MRI sequences	ceT1	N.R.	iPlan software	Excluding enhancing rim

Abbreviations: MRI = magnetic resonance imaging, ce = contrast-enhanced, DWI = diffusion-weighted images, FLAIR = fluid-attenuated inversion recovery, MPRAGE = magnetization-prepared rapid gradient-echo, Gd-DTPA = gadolinium diethylenetriamine penta-acetic acid, TDT = thermal damage threshold, TDE = thermal damage estimate, IPA = immediate post-ablation, FU = follow-up, N.R. = not reported, N.A. = not applicable

Table B: Study results for methods used for calculation of ablated volume or percentage of ablation for epilepsy papers

Author	Year	Imaging modality	Volume Estimation Modality	Timing	Software	In- or exclusion of enhancing rim
Alexander	2019	Conventional MRI sequences	ceT1	IPA	OsiriX DICOM viewer	Including enhancing rim
Aung	2023	Conventional MRI sequences	ce	+ 24 hours	iPlan software	N.R.
Donos	2018	Conventional MRI sequences	ceT1	IPA	FreeSurfer	N.R.
Gadgil	2019	Conventional MRI sequences	ceT1 and DWI	IPA	BrainLab	N.R.
Grewal	2019	Conventional MRI sequences	ceT1	IPA	Analyze 12.0	N.R.
Gupta	2020	Conventional MRI sequences	ceT1	IPA	ITK-SNAP	N.R.
Hwang	2022	Conventional MRI sequences	ceT1	IPA	ITK-SNAP and FreeSurfer	N.R.

Hwang	2022	Conventional MRI sequences	ceT1	IPA	ITK-SNAP	N.R.
Ibrahim	2018	Conventional MRI sequences	T1	N.R.	ITK-SNAP	N.A.
Infante	2024	Conventional MRI sequences	ce	N.R.	N.R.	*"The internal ring of enhancement was considered"*
Jermakowicz	2018	Conventional and thermal MRI sequences	ceT1 MPRAGE and Thermal dose (TDE)	IPA and +6 months	CranialSuite	Including enhancing rim
Jermakowicz	2017	Conventional MRI sequences	MPRAGE	+6 months	Analyze 12.0 and CranialSuite	N.R.
Kang	2016	Conventional MRI sequences	ceT1	IPA	FreeSurfer, MatLab, ITK-SNAP	Including enhancing rim (from figure)
Kim	2021	Conventional MRI sequences	MPRAGE T1 ce	IPA	Freesurfer, ITK-SNAP	N.R.

Kim	2023	Conventional MRI sequences	MPRAGE T1 ce	IPA	FreeSurfer, ITK-SNAP	Including enhancing rim
Kim	2022	Conventional MRI sequences	MPRAGE T1 ce	IPA	FreeSurfer, ITK-SNAP	Including enhancing rim
Ko	2022	Conventional MRI sequences	ceT1	IPA	FreeSurfer	Including enhancing rim
Lombardi	2023	Conventional MRI sequences	DWI T2	IPA	N.R.	DWI with and without rim
Malcolm	2021	Conventional MRI sequences	ceT1 and T2	N.R. for ablation volumes. Long-term FU (resp +7, +6, +12, +5 months) for changes in volumes.	Horos	Including enhancing rim
McCracken	2016	Conventional MRI sequences	ceT1	IPA	BrainLab	N.R.
Mithani	2021	Conventional MRI sequences	ceT1	IPA	ITK-SNAP	N.R.

Morris	2017	Conventional MRI sequences	ceT1	IPA and in "postoperative period"	OsiriX	Including enhancing rim
Ordaz	2023	Conventional MRI sequences	ceT1	N.R.	N.R.	N.R.
Satzer	2020	Conventional MRI sequences	ceT1 (IPA) and T2-hypointensity (FU)	IPA and last follow-up (mean 6 months post-operative)	N.R.	Including enhancing rim (from figure)
Satzer	2021	Conventional MRI sequences	ceT1	IPA	ITK-SNAP and ImageJ	Including enhancing rim
Shofty	2021	Conventional MRI sequences	ceT1	N.R.	Iplannet planning software	N.R.
Slingerland	2023	Conventional MRI sequences	ce volumetric spoiled gradient echo or T1	IPA	Synapse 3D	N.R.
Tao	2020	Conventional MRI sequences	ceT1	IPA	StealthStation	Including enhancing rim

Tao	2018	Conventional MRI sequences	ceT1	IPA	Precis	Including enhancing rim
Willie	2014	Conventional MRI sequences	ceT1	IPA	N.R.	N.R.
Willie	2019	Conventional MRI sequences	ceT1 (IPA) and T2 (last FU)	IPA and last available follow-up MRI (mean 18 ± 10 months)	OsiriX	Including enhancing rim
Wu	2015	Conventional MRI sequences	N.R.	IPA	FreeSurfer, ITK-SNAP and MatLab	N.A.
Zheng	2023	Conventional MRI sequences	ceT1	IPA	AFNI	Including enhancing rim

Abbreviations: MRI = magnetic resonance imaging, ce = contrast-enhanced, DWI = diffusion-weighted images, FLAIR = fluid-attenuated inversion recovery, MPRAGE = magnetization-prepared rapid gradient-echo, Gd-DTPA = gadolinium diethylenetriamine penta-acetic acid, TDT = thermal damage threshold, TDE = thermal damage estimate, IPA = immediate post-ablation, FU = follow-up, N.R. = not reported, N.A. = not applicable

Table C: Study results for methods used for calculation of ablated volume or percentage of ablation for combined oncology and epilepsy and/or other indications papers

Author	Year	Indication	Imaging modality	Volume Estimation Modality	Timing	Software	In- or exclusion of enhancing rim
Attaar	2015	Oncology, epilepsy and pain	Conventional MRI sequences	N.R.	*"Post-operative"*	IMPAX	Excluding enhancing rim
Dadey	2016	Oncology and epilepsy	Thermal MRI sequences	Thermal dose (TDT line)	IPA	NeuroBlate	N.A.
Jensdottir	2023	Oncology and epilepsy	Conventional MRI sequences	ceT1	N.R.	BrainLab	Excluding enhancing rim
Laurent	2018	Oncology and epilepsy	Conventional MRI sequences	ceT1	N.R.	In-house computer graphics workstation and MatLab	Including enhancing rim (from figure)
Liang	2021	Oncology and epilepsy	Thermal MRI sequences	Thermal dose (in-house TDE model)	IPA	MatLab	N.A.

Abbreviations: MRI = magnetic resonance imaging, ce = contrast-enhanced, MPRAGE = magnetization-prepared rapid gradient-echo, TDT = thermal damage threshold, IPA = immediate post-ablation, N.R. = not reported., N.A. = not applicable
